# Synthesizing multi-frame high-resolution fluorescein angiography images from retinal fundus images using generative adversarial networks

**DOI:** 10.1186/s12938-023-01070-6

**Published:** 2023-02-21

**Authors:** Ping Li, Yi He, Pinghe Wang, Jing Wang, Guohua Shi, Yiwei Chen

**Affiliations:** 1 School of Optoelectronic Science and Engineering, University of Electronic Science and Technology of China, Chengdu, 611731 China; 2 Suzhou Institute of Biomedical Engineering and Technology, Chinese Academy of Sciences, Suzhou, 215163 China; 3 School of Biomedical Engineering (Suzhou), Division of Life Sciences and Medicine, University of Science and Technology of China, Hefei, 230026 China

**Keywords:** Retinal fundus images, Fluorescein angiography images, Multi-frame, High-resolution, Generative adversarial network

## Abstract

**Background:**

Fundus fluorescein angiography (FA) can be used to diagnose fundus diseases by observing dynamic fluorescein changes that reflect vascular circulation in the fundus. As FA may pose a risk to patients, generative adversarial networks have been used to convert retinal fundus images into fluorescein angiography images. However, the available methods focus on generating FA images of a single phase, and the resolution of the generated FA images is low, being unsuitable for accurately diagnosing fundus diseases.

**Methods:**

We propose a network that generates multi-frame high-resolution FA images. This network consists of a low-resolution GAN (LrGAN) and a high-resolution GAN (HrGAN), where LrGAN generates low-resolution and full-size FA images with global intensity information, HrGAN takes the FA images generated by LrGAN as input to generate multi-frame high-resolution FA patches. Finally, the FA patches are merged into full-size FA images.

**Results:**

Our approach combines supervised and unsupervised learning methods and achieves better quantitative and qualitative results than using either method alone. Structural similarity (SSIM), normalized cross-correlation (NCC) and peak signal-to-noise ratio (PSNR) were used as quantitative metrics to evaluate the performance of the proposed method. The experimental results show that our method achieves better quantitative results with structural similarity of 0.7126, normalized cross-correlation of 0.6799, and peak signal-to-noise ratio of 15.77. In addition, ablation experiments also demonstrate that using a shared encoder and residual channel attention module in HrGAN is helpful for the generation of high-resolution images.

**Conclusions:**

Overall, our method has higher performance for generating retinal vessel details and leaky structures in multiple critical phases, showing a promising clinical diagnostic value.

**Supplementary Information:**

The online version contains supplementary material available at 10.1186/s12938-023-01070-6.

## Background

Many disease-related biomarkers can be observed from fundus images, such as optic disc, optic cup, macula, blood vessels, hemorrhages, exudates, and microaneurysms. When compared with traditional methods of designing features manually, deep learning can automatically learn features from data. Many studies have used deep learning for lesion segmentation, disease classification and image synthesis of fundus images.

In terms of segmentation, Dai et al. [1] proposed a multi-sieving convolutional neural network based on the clinical reports to detect microaneurysms. Guo et al. [2] proposed a bin loss and a top-k loss to improve exudate segmentation performance. Yan et al. [3] proposed a three-stage model to solve the imbalance of pixel ratio between thick vessels and thin vessels, including thick vessel segmentation, thin vessel segmentation and vessel fusion. Wang et al. [4] proposed a coarse-to-fine supervised network for vessel segmentation and used a feature augmentation module to improve vessel segmentation performance. Fu et al. [5] proposed the M-Net that can segment the optic cup and optic disk in one stage. In M-Net, multi-scale input, lateral output, and multi-label loss function are used to accurately separate the optic disc and optic cup. Fu's method greatly inspired later work on the segmentation of optic disks and optic cups. Wang et al. [6] proposed the patch-based Output Space Adversarial Learning framework, which encourages the segmentation similarity between the source domain and the target domain to solve the challenge of the domain transfer. The authors also propose a novel morphology-aware loss that guides precise optic disc and cup segmentation. Liu et al. [7] proposed a semi-supervised segmentation GAN, which consists of a segmentation network, a generator and a discriminator. Segmentation networks can adopt samples from mixed labeled and unlabeled data in a semi-supervised manner. A good segmentation of the optic disc and cup can be achieved with a small amount of labeled data.

In terms of classification, Ahmad et al. [8] conducted benchmarking work on the Messidor-2 dataset, evaluating eight deep-learning classification models and generating CAMs for lesions simultaneously. The results show that with the increase of network depth and parameters, the classification performance will be better, but the location performance will be worse. To build an optimal diabetic retinopathy classification model, Zhang et al. [9] established a high-quality labeled dataset, combined popular neural networks using an ensemble strategy, explored the relationship between the number of classifiers and the number of class tags, as well as the effect of different combinations of classifiers on performance. Grassmann [10] divided age-related macular degeneration into 13 classes, trained the images independently using 6 different CNNs, and finally fused the results of the 6 networks using random forest. Wang et al. [11] used a multi-task learning model to diagnose 36 diseases simultaneously, and their network structure has two stages. In the first stage, there is an improved YOLO-v3 to detect the macula and the optic disc area. In the second stage, there are 3 branches, which are used to detect general retinal diseases, macular-related diseases, and optic disc-related diseases.

Fundus image synthesis has been widely used in two aspects. Firstly, it is difficult to obtain a large number of high-quality medical image data, so it is a good solution to expand the dataset by generating adversarial network to generate images [12–14]. On the other hand, image conversion is one of the important applications of image synthesis. Image can be converted from one domain to another by using the generative adversarial network, which has been applied well in MRI to CT [15–17]. This study also belongs to this field, which is to convert fundus images into fluorescein angiography (FA) images.

FA is a standard diagnostic tool for fundus diseases, which allows dynamic observation of retinal vascular circulation using fluorescein under physiological and pathological conditions [18]. FA can be divided into prefilling, transit, recirculation, and late phases. In the transit and recirculation phases, the filling state and time of fluorescein in retinal blood vessels are essential parameters for the diagnosis of retinal vascular occlusive diseases. In the late phase, abnormal lesions have maximal contrast as fluorescein fades, which is critical for the diagnosis of retinal-associated hemangiomas and diabetes [19, 20]. Angiography is an invasive procedure that requires the injection of fluorescein, which may cause some adverse reactions in patients allergic to fluorescein [21, 22]. Alternatively, FA images of multiple critical phases can be generated from retinal fundus images for diagnosis while avoiding risk to patients.

The generation of FA images from retinal fundus images can be formulated as an image transformation problem, which can be suitably solved using deep learning or generative adversarial network (GAN) [23–25]. Hervella et al. [26] constructed a U-Net to directly learn the relations between retinography and FA images. Schiffers et al. [27] used a CycleGAN to achieve the unsupervised synthesis of fundus FA images. Li et al. [28] proposed a pixel-to-pixel approach for the supervised synthesis of fundus FA images. However, the abovementioned methods can only generate single-phase FA images. Li et al. [29] recently proposed SequenceGAN with multiple generators and discriminators to generate FA images of multiple phases from retinal fundus images. However, the generated images are of low resolution because the multiple generators are demanding in terms of computations and memory, and the discriminator easily distinguishes synthetic and real images at high resolution, consequently hindering training. Kamran et al. [30] proposed Attention2AngioGAN comprising rough and fine generators to handle problems related to high-resolution images. Attention2AngioGAN allows to generate single-frame FA images of 512 × 512 pixels. However, training requires 16 GB of memory on the professional NVIDIA Tesla P100 graphics card, thus requiring expensive specialized hardware to generate multi-frame high-resolution FA images. Patching/splicing can be used to overcome memory limitations for image generation [31], but the patches are independently trained and lack global intensity information. Even if overlapping and weighted fusion are adopted for splicing, details are lost, and blurry images are generated [32].

Overall, the existing methods generate low-resolution or single-frame FA images, which may be unsuitable for the diagnosis of fundus diseases. Therefore, a method that achieves high-quality image generation performance of multiple key phases must be developed. We propose a method to generate multi-frame high-resolution FA images from retinal fundus images. Our main contributions are as follows.

First, we combined unsupervised and supervised learning to generate full-size and high-resolution FA images. Our framework consists of an LrGAN for generating low-resolution fundus fluorescence images and an HrGAN for generating high-resolution multi-frame FA images.

Second, we propose a shared encoder, which is trained by iteratively extracting FA image features of three phases to ensure the performance of the encoder.

Finally, our experimental results demonstrate better performance in generating vascular structure and leakage details as compared to classical unsupervised and supervised learning methods, thus can better assist physicians in diagnosis. Quantitative and qualitative comparisons between our method and available methods show the superiority of our proposal.

## Results

We conducted various experiments in a Linux environment with Python 3.6. We trained the model for 250 epochs. We use Adam with momentum values ${\beta }_{1}$ = 0.5 and ${\beta }_{2}$ = 0.999, and learning rate $l$ = 0.0002 as the optimizer. It took approximately 57 h to train the model on a computer equipped with an NVIDIA Tesla P100 graphics card.

### Datasets and implementation details

**Fig. 1:**
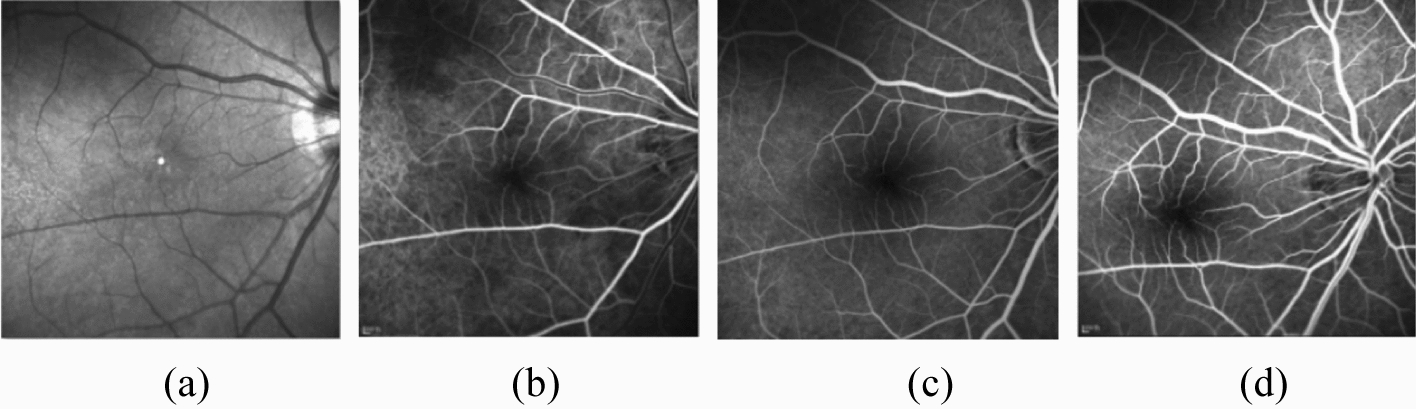
Illustration of the types of image: **a** fundus structure image, **b** the transit-phase FA image, **c** the recirculation-phase FA image, and **d** the late-phase FA image

In this study, the dataset was collected from the Third People's Hospital of Changzhou using a Heidelberg confocal fundus angiography system between March 2011 and September 2019. The dataset includes images of 252 eyes from 216 patients (92 women and 124 men aged 17–72 years). Each image pair includes a fundus structure image of 768 × 768 pixels and three corresponding FA images of 768 × 768 pixels from the three phases (Fig. 1). The collected fundus structure and FA images are not aligned in general. From the image pairs, 126 were randomly chosen for LrGAN, and the remaining 126 were selected for HrGAN.

For LrGAN, unsupervised learning was applied to generate low-resolution FA images of 768 × 768 pixels. We finally obtained 424 fundus structure images and 1272 FA images by data augmentation, including rotation and flipping. The fundus structure images will be used as input for the first generator in LRGAN, and the FA images will be used as input for the second generator in LRGAN.

For HrGAN, supervised learning was applied to generate high-resolution FA image patches, and the inputs and outputs of the network were strictly aligned. Therefore, we used the image registration method in [28] to process the non-aligned fundus structure and FA images of 768 × 768 pixels, from which we obtained aligned images of 400 × 400 pixels. Then, we randomly cropped the aligned images to obtain patches of 256 × 256 pixels, obtaining 126 patch pairs for testing and 2788 patch pairs for training, where the fundus structure images and the low-resolution FA images generated by LRGAN were used as inputs, and the real FA images of three phases were used as outputs.

### Qualitative evaluation

To demonstrate the effectiveness of the proposed method, we compared it with HrGAN, the methods in [29] and [33], StarGAN [34], VtGAN [23], BicycleGAN [35], and Unet [26]. The method in [33] performs one-to-one image transformation, whereas StarGAN performs one-to-many image transformation, and both methods are unsupervised. The methods in [29], VtGAN, BicycleGAN, and Unet are supervised.

**Fig. 2:**
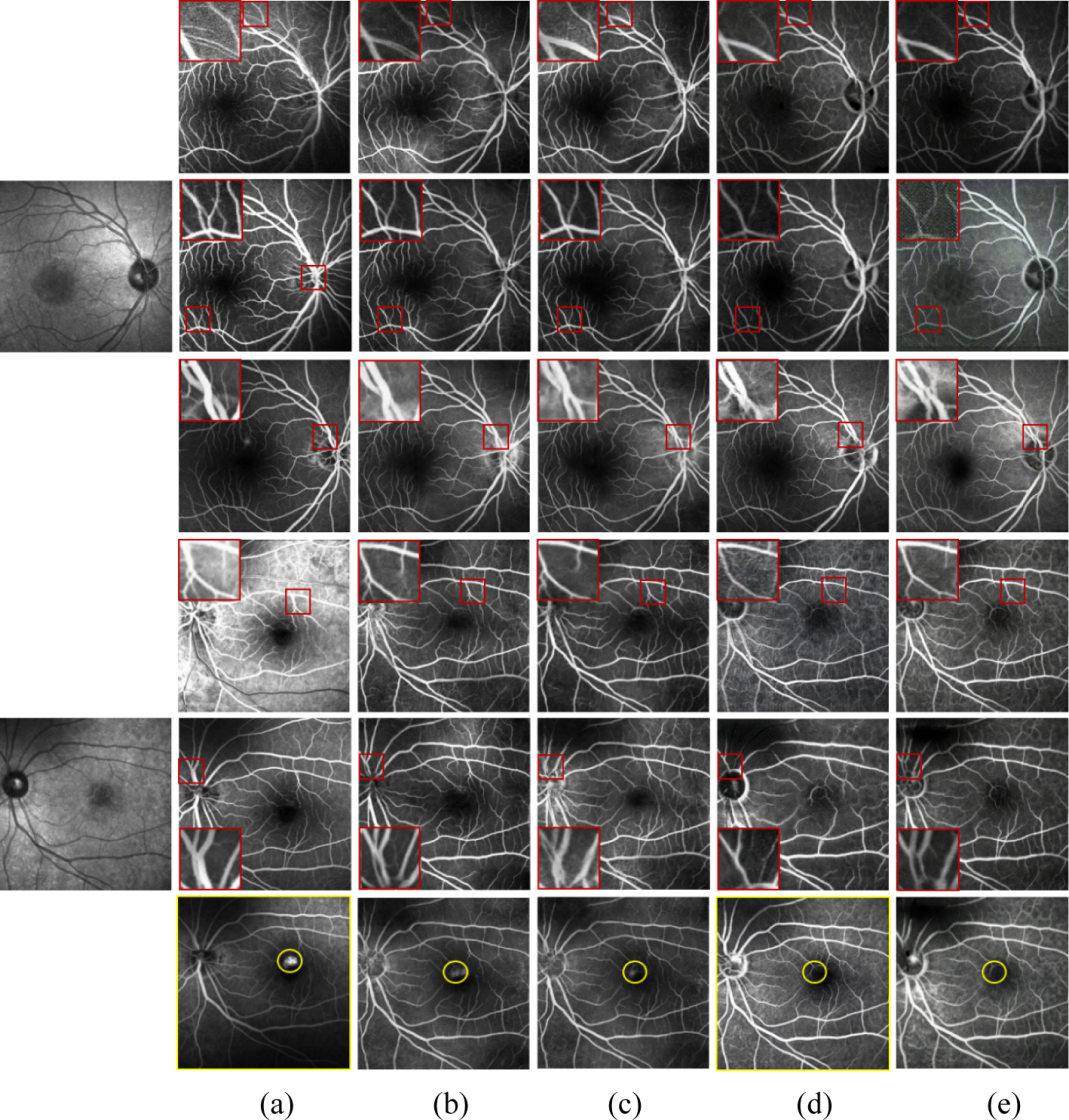
Results of state-of-the-art unsupervised methods for FA image generation. The first to third and fourth to sixth rows show the transit phase to the late phase FA images, respectively, and the first column shows the original fundus structure image. The fundus structure and generated FA images have a size of 768 × 768 pixels. For a better comparison, we magnified the area enclosed in the red box. **a** Real FA image and results of **b** proposed method (LrGAN + HrGAN), **c** HrGAN, **d** method in [33], and **e** StarGAN

Figure 2 shows the results of the qualitative comparison with state-of-the-art unsupervised methods. As shown in Fig. 2d–e, the FA images generated by the method of [33] and the method of StarGAN show the basic vascular structure, where some fine vessels are not generated, and the brightness of the generated FA images is different from the real FA images. As shown in Fig. 2c, without the low-resolution FA images generated by LrGAN as input, the FA images generated by HrGAN lose details and appear blurred in the regions with dense blood vessels. When compared with the existing unsupervised methods of HrGAN, the method in [33], and StarGAN, the proposed method generates the most similar images to the real FA images, showing leakage and fine vessels (Fig. 2b).

**Fig. 3:**
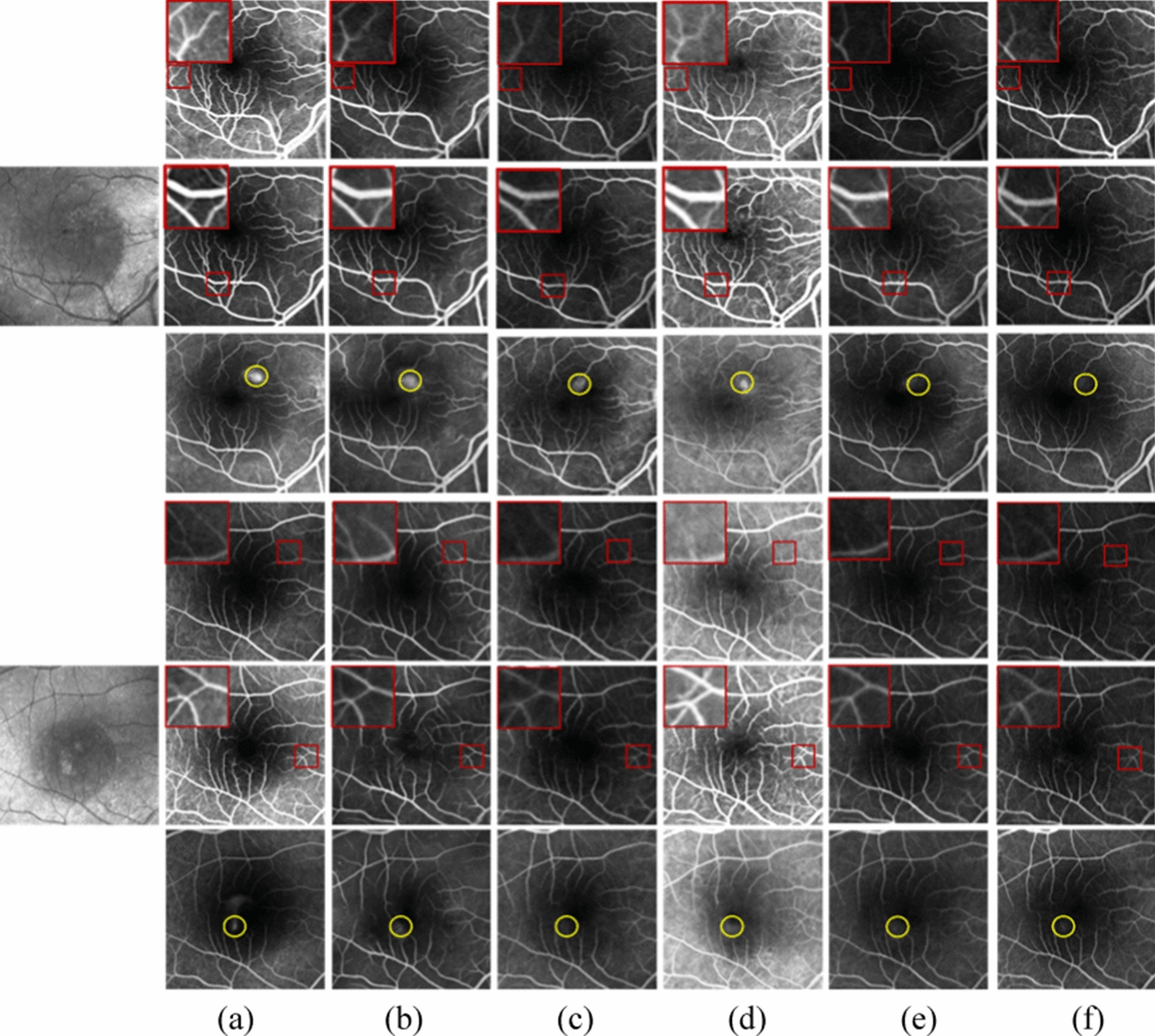
Results of state-of-the-art supervised methods. The first to third and fourth to sixth rows show the transit phase to the late phase FA images, respectively, and the first column shows the fundus structure image. **a** Real FA image and results of **b** proposed method (LrGAN + HrGAN), **c** method in [29], **d** BicycleGAN, **e** VtGAN, and **f** Unet

Figure 3 shows the results of the qualitative comparison with state-of-the-art supervised methods that generate FA images of 400 × 400 pixels. When compared with the unsupervised generation methods of HrGAN, the method in [33], and StarGAN, the supervised methods in [29], BicycleGAN, and VtGAN produce better visual effects images, as shown in Fig. 3c–e. This is because high-resolution images are more difficult to train. Supervised methods require aligned images, and the images collected in hospitals are often non-aligned owing to equipment, eye shaking, and other factors. After alignment, the image size is notably reduced. As shown in Fig. 3c, the method of [29] fails to generate blood vessels clearly in image regions with low brightness. Figure 3d shows the FA image generated by BicycleGAN. We can see that there is noise in the generated FA image and some fine vessels blending with the surrounding area, making observation difficult. When compared with the generation of adversarial network, Unet lacks adversarial learning. In addition, the number and diversity of data is not enough, so supervised learning is more likely to cause overfitting, thus reducing the generalization ability of the model. From Fig. 3f, we can see that the Unet does not capture the leakage, and even some leakage becomes part of the vasculature. Figure 3b shows that our method achieves comparable results to supervised methods for generating FA images.

### Quantitative evaluation

For quantitative evaluation, we used common indicators, including structural similarity (SSIM) [36], normalized cross-correlation (NCC) and peak signal-to-noise ratio (PSNR) [37], that measure the similarity between the real FA images and the generated FA images. The PSNR, SSIM and NCC are given by

1 $$MSE=\frac{\sum_{n=1}^{N}({x}^{n}-{y}^{n})}{N}$$

2 $$PSNR=10\times \mathrm{log}\left(\frac{{255}^{2}}{MSE}\right)$$

3 $$SSIM\left(x,y\right)=\frac{\left(2{\mu }_{x}{\mu }_{y}+{c}_{1}\right)\left(2{\sigma }_{xy}+{c}_{2}\right)}{\left({\mu }_{x}^{2}+{\mu }_{y}^{2}+{c}_{1}\right)\left({\sigma }_{x}^{2}+{\sigma }_{y}^{2}+{c}_{2}\right)}$$

4 $$NCC\left(x,y\right)=\frac{{\sigma }_{xy}}{{\sigma }_{x}{\sigma }_{y}}$$

where $x$ and $y$ are the generated image and the actual image. $n$ and $N$ are the nth pixel and the image size, respectively. ${\mu }_{x}$, ${\sigma }_{x}$ and ${\sigma }_{xy}$ are the mean standard deviation and covariance, respectively.

Table 1 lists the evaluation results for FA images generated by HrGAN, the method in [33], StarGAN, the method in [29], VtGAN, BicycleGAN, and our method (LrGAN + HrGAN) in terms of SSIM, PSNR, and NCC.

**Table 1:** Performance evaluation of FA image generation methods

	HrGAN	Hervella’s method	Star GAN	Li’s method	Bicycle GAN	VtGAN	Unet	Our method
SSIM	0.6924	0.7081	0.6013	**0.7194**	0.6781	0.7034	0.6840	0.7126
PSNR(dB)	15.67	15.50	12.50	15.63	13.71	15.60	15.63	**15.77**
NCC	0.7421	0.6573	0.5621	0.7393	0.6901	0.7085	0.7030	**0.7699**

The bold data represent the best value obtained

The average SSIM of our method is 0.0202, 0.0045, and 0.1113 higher than that of the unsupervised methods of HrGAN, the method in [33], and StarGAN, respectively. The average PSNR improvements are 0.1, 0.27, and 3.27, respectively. The average NCC improvements are 0.0278, 0.1126, and 0.2078, respectively. Compared with supervised methods, our method achieves the best indicators (SSIM of 0.7126, PSNR of 15.77 and NCC of 0.7699). Hence, our methods can able to provide higher-quality FA images than the existing methods.

To determine the confidence of the results, we asked two ophthalmologists to assess the quality of the generated FA images. We randomly selected 50 images from the test set, 25 of which were true and 25 of which were false. The ophthalmologists were not aware of the authenticity of the images during the experiment. Table 2 shows the detailed results of the identification. According to Table 2, it can be seen that the experts identified 76% of the generated images as real, while 88% of the real images were also identified as real. Although the precision was only 46.3%, the images produced by our model managed to fool eye specialists.

**Table 2:** The results of the experiment are verified by an ophthalmologist

	Results		Average
	Fake (%)	Real (%)	Precision (%)
Fake	24	76	46.3
Real	88	12	

### Ablation study

We quantitatively compare the proposed model with the model’s baseline to verify the detail branch’s effect. The baseline of the model is HrGAN without patch, residual attention block, common decoder, perceptual loss and feature matching loss.

As shown in Table 3, we can see that the patch strategy is much better than the unsupervised approach on PSNR. Furthermore, we can find that the residual attention block has the greatest effect on model improvement. In addition, low-resolution FA image generated by LrGAN as HrGAN input is helpful to improve the generated results.

**Table 3:** Results of the proposed model’s ablation study

Base	Lrgan	Patch	Res	${G}_{e}$	${L}_{FM}$	${L}_{percep}$	PSNR	NCC
√	√						13.71	0.6901
√		√					14.92	0.6813
√		√			√	√	15.05	0.6824
√		√		√	√	√	15.29	0.7082
√		√	√	√	√	√	15.67	0.7421
√	√	√	√	√	√	√	**15.77**	**0.7699**

The bold data represent the best value obtained

## Discussion

It should be noted that the fundus structure images and the FA images of the three phases in our datasets are often not aligned. Therefore, both unsupervised and supervised learning methods have limitations in generating FA images. The unsupervised learning method does not require the input and output to be aligned, but this method can only roughly generate low-resolution FA images and cannot accurately generate vascular structures and leakage areas, which are essential for the physician's diagnosis. Supervised learning methods require the input and output to be aligned one-to-one, but this method significantly reduces the field of view of FA images. Therefore, we designed two GANS to generate high-resolution quality images. LrGAN generated low-resolution and full-size imaging images with global intensity information, and HRGAN generated high-resolution and multi-frame imaging patches and merged full-size images. In HRGAN, we use a shared encoder among multiple generators so that FA images of different periods can be utilized to make the encoder more capable of extracting features. In addition, we use the residual channel attention module in the decoder to give different weights to each channel in the feature space so that the network can learn the details in the image more effectively and generate high-quality images. In addition to the above two points, we introduce pixel loss, feature matching loss, and perception loss to make the low-level details and high-level semantic features of the image generated by the network as consistent as possible with the original image.

Our method can achieve the expected results, but the proposed method requires two GANS containing multiple generators to generate multi-frame high-resolution FA images with a long training time. We hope to simplify the model in future work to generate high-quality FA images in one stage. In addition, the model does not capture the micro-leakage very well, and we hope to solve this problem through multi-scale network learning.

## Conclusions

Fundus FA is a common imaging method for diagnosing fundus diseases, but poses potential risks to patients. GANs have enabled the generation of FA images from fundus structure images. However, the existing GANs can only generate single-frame/low-resolution FA images, which are unsuitable for correct diagnosis. The proposed method of LrGAN + HrGAN can generate multi-frame high-resolution FA images from fundus structure images. Our method can provide high-quality FA images compared to unsupervised methods. In addition, our method can generate high-resolution FA images than supervised methods. Furthermore, the proposed method can generate FA images of various critical phases. In conclusion, our method has higher overall performance for generating retinal vessel details and leaky structures in multiple critical phases, showing a promising clinical diagnostic value. In the future, the proposed model can be further studied to simplify and improve the performance in detail generation.

## Methods

### Flowchart of our approach

**Fig. 4:**
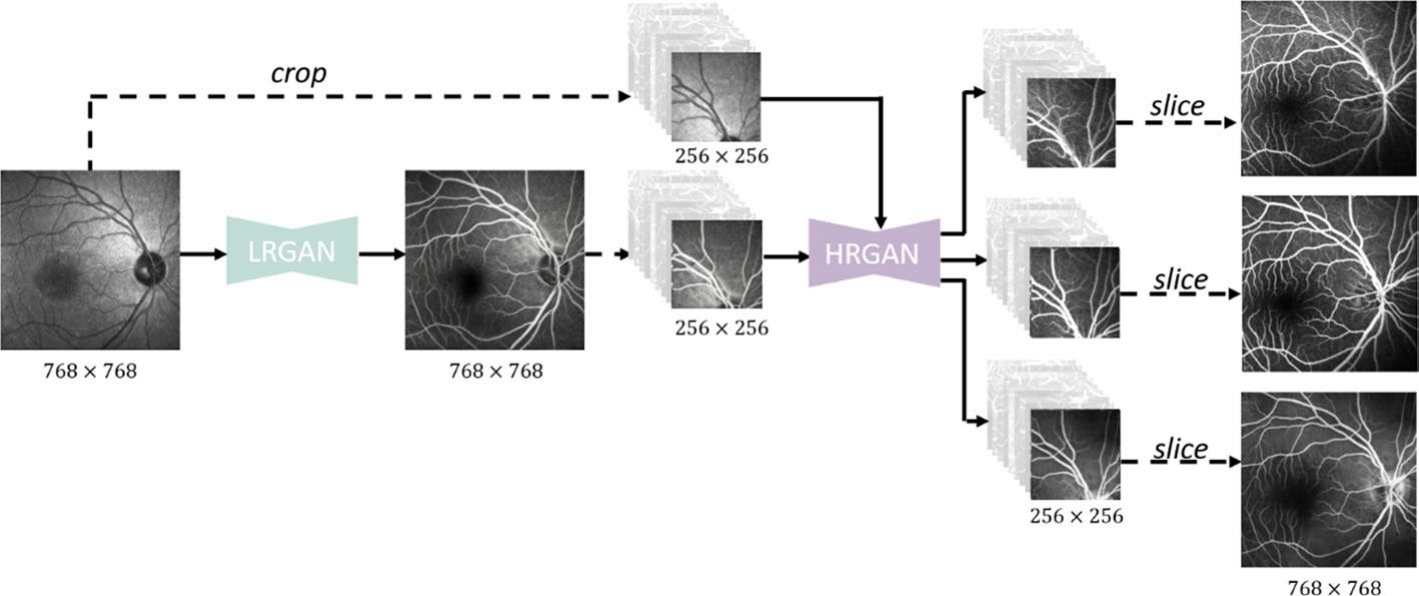
Flowchart of the proposed method for generating multi-frame high-resolution FA images

A flowchart of the proposed method for generating multi-frame high-resolution FA images is shown in Fig. 4. We first train the LrGAN to generate a low-quality FA image of 768 × 768 pixels from a fundus structure image of the same size. Next, the FA image is cropped along with the fundus structure image into images of 256 × 256 pixels. Then, they are input into the HrGAN based on the multiple generators and discriminators to obtain high-quality FA image patches of 256 × 256 pixels. Finally, using weighted fusion, we merge the FA image patches of 256 × 256 pixels into a high-resolution FA image of 768 × 768 pixels.

### LrGAN for low-resolution FA images

**Fig. 5:**
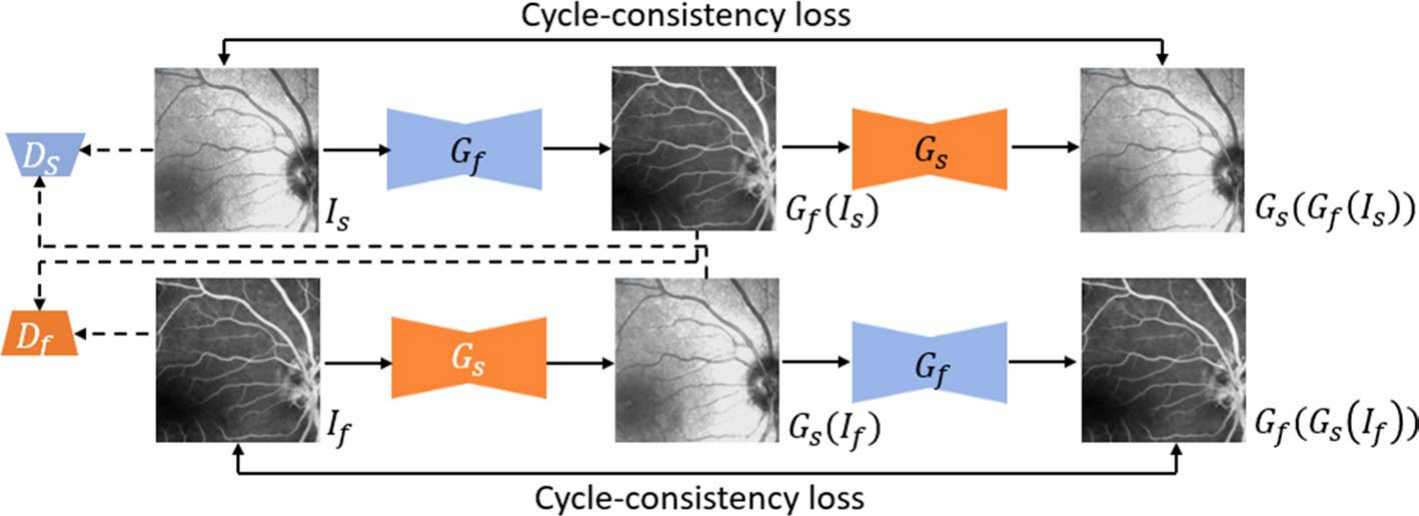
Architecture of proposed LrGAN. Generators ${G}_{f}$ and ${G}_{s}$ and discriminators ${D}_{s}$ and ${D}_{f}$ are used to generate FA and fundus structure images

To generate low-resolution FA images that retain the global intensity of fundus structure images, we introduce LrGAN based on the CycleGAN [38]. LrGAN consists of two generators and two discriminators, as shown in Fig. 5. Generator ${G}_{f}$ provides FA images from fundus structure images, and generator ${G}_{s}$ converts FA images into fundus structure images. The two discriminators, ${D}_{f}$ and ${D}_{s}$, are intended to determine the authenticity of the generated images. Owing to memory limitations, we use 70 × 70 PatchGAN [39] as the discriminator and six residual blocks [40] as the generator.

We use cycle-consistency loss ${L}_{\mathrm{CC}}$ and adversarial loss ${L}_{\mathrm{GAN}}$ in LrGAN to generate low-resolution FA images. The objective function is the combination of ${L}_{\mathrm{CC}}$ and ${L}_{\mathrm{GAN}}$ as follows:

5 $$L={\alpha L}_{GAN}+\beta {L}_{CC}$$

where $\alpha$ and $\beta$ are hyperparameters determined experimentally to control the contributions of ${L}_{GAN}$ and ${L}_{CC}$, respectively. After evaluating these parameters, we set $\alpha =1$ and $\beta =10$ to achieve suitable performance.

The adversarial loss is given by

6 $$L_{GAN} = E\left[ {{\text{log}}D_{s} \left( {I_{S} } \right)} \right] + E\left[ {{\text{log}}\left( {1 - D_{S} \left( {G_{S} \left( {I_{f} } \right)} \right)} \right)} \right]\, + \,E\left[ {{\text{log}}D_{f} \left( {I_{f} } \right)} \right] + E\left[ {{\text{log}}\left( {1 - D_{f} \left( {G_{f} \left( {I_{s} } \right)} \right)} \right)} \right]$$

and the cycle-consistency loss is given by

7 $$L_{CC} = E\left[ {\parallel G_{f} \left( {G_{s} \left( {I_{f} } \right)} \right) - I_{f} \parallel_{1} \left] { + E} \right[\parallel G_{s} \left( {G_{f} \left( {I_{s} } \right)} \right) - I_{s} \parallel_{1} } \right]$$

Whether unsupervised GAN or supervised GAN, using only one GAN to generate FA images has limitations. Unsupervised GAN does not require the input and output to be aligned, but this method can only generate low-resolution FA images roughly and cannot accurately generate vascular structures and leakage areas, which are essential for physicians' diagnosis. Supervised GAN requires that the inputs and outputs are aligned, but the fundus structure images and FA images in our dataset are often not strictly aligned. Therefore, it is necessary to register the structure and FA images first, crop them to the same size, and then input them to supervised GAN. After the above operations, the field of view of the FA image will be significantly reduced. We can merge the full-size image with patches, but we may lose detail or even blur at the boundaries because the patches are generated independently and lack global intensity information between them. Therefore, we hope the inputs in supervised GAN will also have global intensity information, so we build the LrGAN to generate low-resolution FA images with global information as part of the input to HrGAN.

### HrGAN for high-resolution FA images patches

**Fig. 6:**
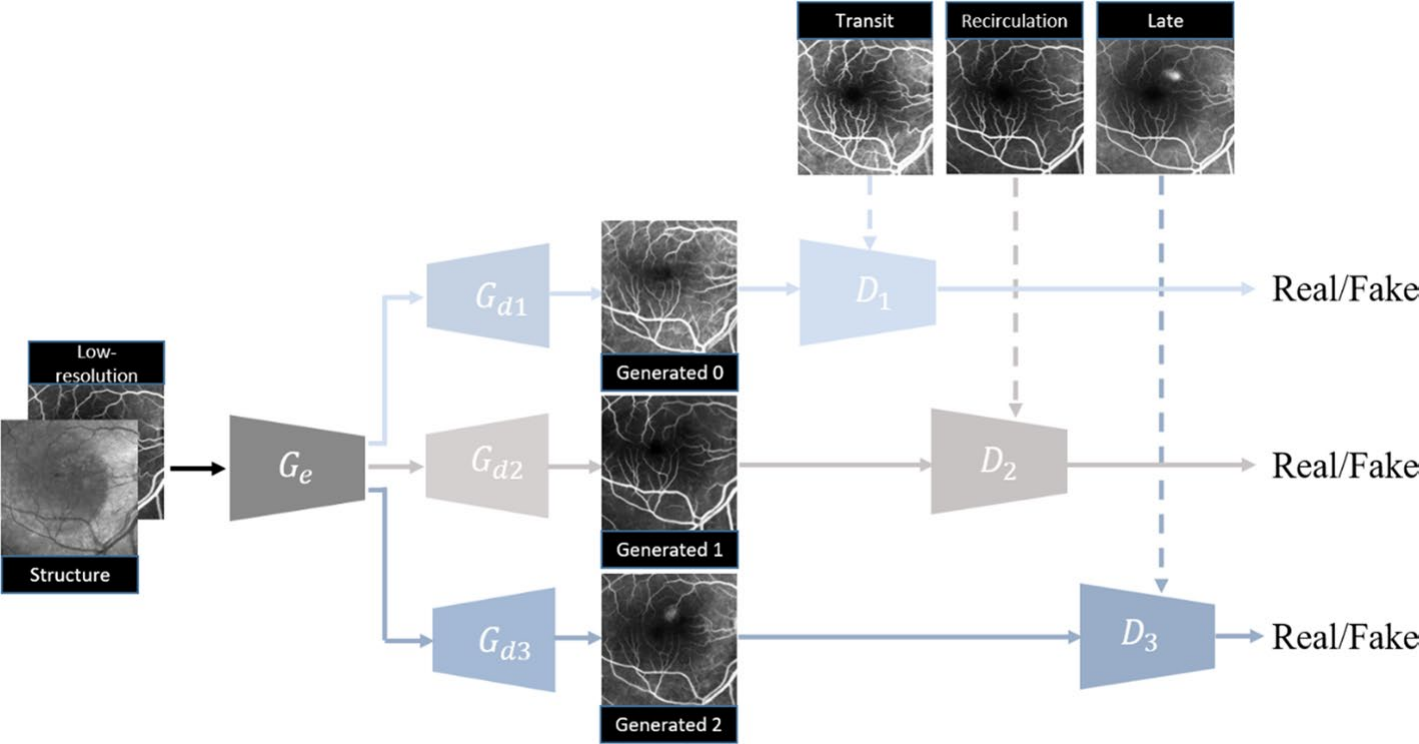
Architecture of proposed HrGAN with common encoders ${G}_{e}$, decoders ${G}_{d1}$, ${G}_{d2}$, and ${G}_{d3}$, and discriminators ${D}_{1}$, ${D}_{2}$, and ${D}_{3}$. ${G}_{e}$, ${G}_{d1}$, and ${D}_{1}$ are used for transit-phase FA image generation, while ${G}_{e}$, ${G}_{d2}$, and ${D}_{2}$ are used for recirculation-phase FA image generation, and ${G}_{e}$, ${G}_{d3}$, and ${D}_{3}$ are used for transit-phase FA image generation

**Fig. 7:**
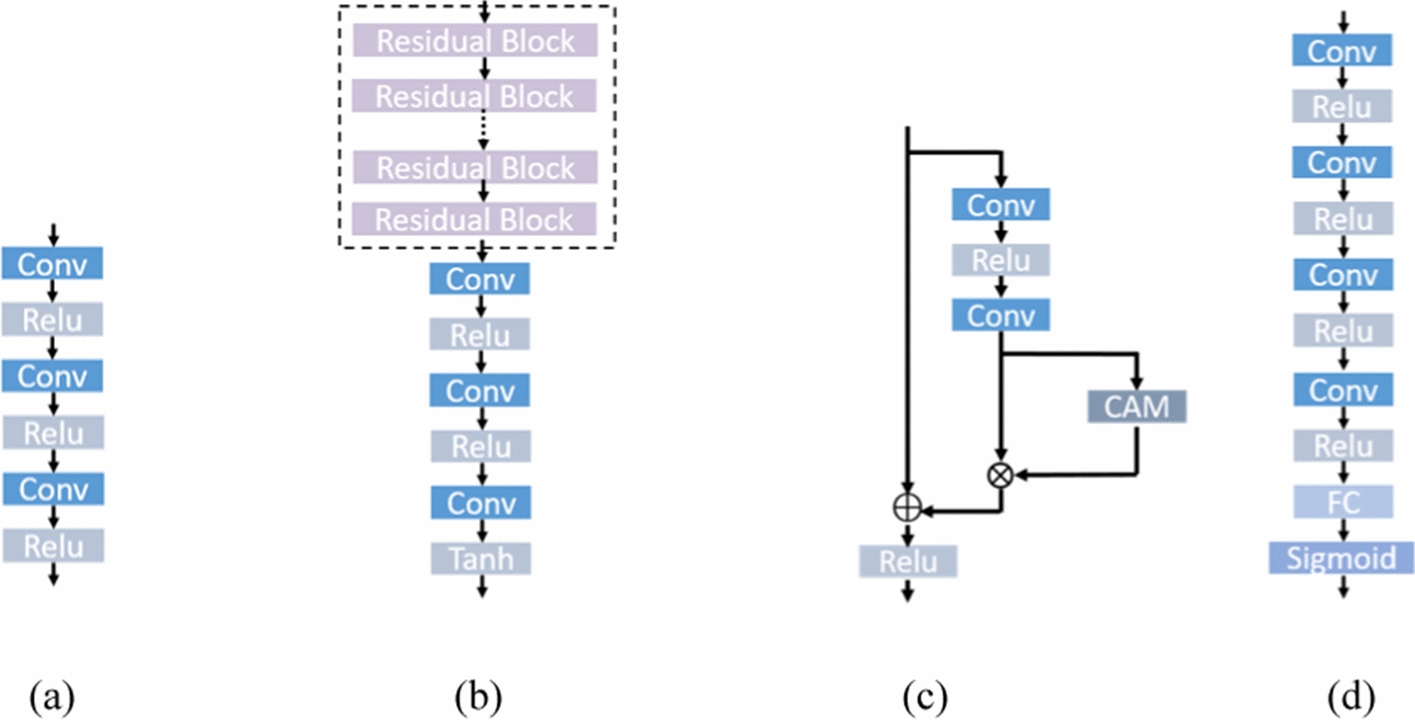
Architecture of generators and discriminators in HrGAN. The architecture of **a** ${G}_{e}$, **b** ${G}_{d1}$,${G}_{d2}$, and ${G}_{d3}$, **c** residual attention block, and **d** ${D}_{1}$, ${D}_{2}$, and ${D}_{3}$. *Conv* convolutional layer, *Relu* rectified linear unit, *CAM* channel-attention module, *Tanh* hyperbolic tangent function, *FC* fully connected layer

To generate fundus FA images from multiple critical phases, the proposed HrGAN has a generator composed of one common encoder, ${G}_{e}$, and three decoders, ${G}_{d1},{G}_{d2}, and {G}_{d3}$, as shown in Fig. 6. Generator ${G}_{e}$ is trained to encode the fundus structure and low-resolution FA image patches to output feature maps: ${G}_{e}$(${I}_{S},{I}_{f}$) → ${I}_{\mathrm{feature}}$. Decoder ${G}_{d1}$ is trained to generate transit-phase FA images from the encoded feature map: ${G}_{d1}({I}_{\mathrm{feature}})$→${I}_{F1}$, and we add six residual attention blocks [41, 42] to the decoder to extract the features of different FA phases, as shown in Fig. 7. Similarly, we generate recirculation- and late-phase FA images from ${G}_{e}$, ${G}_{d2}$ and ${G}_{d3}$: ${G}_{d2}({G}_{e}({I}_{S},{I}_{f}))$→${I}_{F2}$, ${G}_{d3}({G}_{e}({I}_{S},{I}_{f}))$→${I}_{F3}$. Three discriminators, ${D}_{0}$, ${D}_{1}$ and ${D}_{2}$ are used to determine the authenticity of ${I}_{F1}$, ${I}_{F2}$, and ${I}_{F3}$, respectively. Forward propagation is simultaneous, whereas backpropagation sequentially updates the gradients. An additional file shows the training procedure of HrGAN in more detail [see Additional file 1].

To make the generated FA image indistinguishable from the target image, four loss functions are applied in HrGAN: adversarial (${L}_{\mathrm{GAN}}$), pixel-space (${L}_{\mathrm{pixel}}$), perceptual (${L}_{\mathrm{percep}}$), and feature-matching (${L}_{\mathrm{FM}}$) loss functions. The objective function of training is obtained by combining the loss functions as follows:

8 $$L\, = \,\alpha L_{{{\text{GAN}}}} + \beta L_{{{\text{pixel}}}} + \gamma L_{{{\text{percep}}}} + \delta L_{{{\text{FM}}}}$$

where $\mathrm{\alpha }$, $\beta$, $\gamma$, and $\delta$ are hyperparameters determined experimentally to control the contribution of the corresponding loss functions. We performed various experiments to set appropriate parameter values for $\mathrm{\alpha }$, $\beta$, $\gamma$, and $\delta$ of 1, 100, 0.001, and 0.001, respectively.

Because HrGAN includes three generators and three discriminators, the adversarial loss is given by

9 $$L_{{{\text{GAN}}}} = E\left[ {{\text{log}}D_{1} \left( {I_{F1} } \right)} \right] + E\left[ {{\text{log}}\left( {1 - D_{1} \left( {G_{d1} \left( {G_{e} \left( {I_{s} ,I_{f} } \right)} \right)} \right)} \right)} \right]\, + \,E\left[ {{\text{log}}D_{2} \left( {I_{F2} } \right)} \right] + E\left[ {{\text{log}}\left( {1 - D_{2} \left( {G_{d2} \left( {G_{e} \left( {I_{s} ,I_{f} } \right)} \right)} \right)} \right)} \right]\, + \,E\left[ {{\text{log}}D_{3} \left( {I_{F3} } \right)} \right] + E\left[ {{\text{log}}\left( {1 - D_{3} \left( {G_{d3} \left( {G_{e} \left( {I_{s} ,I_{f} } \right)} \right)} \right)} \right)} \right]$$

To make the generated FA image indistinguishable from the real FA image, e in pixel space, we use the $L1$ loss ${L}_{\mathrm{pixel}}$:

10 $$L_{{{\text{pixel}}}} = E\left[ {\parallel G_{d1} \left( {G_{e} \left( {I_{s} ,I_{f1} } \right) - I_{F1} } \right)\parallel_{1} } \right] + E\left[ {\parallel G_{d2} \left( {G_{e} \left( {I_{s} ,I_{f2} } \right) - I_{F2} } \right)\parallel_{1} } \right]\, + \,{ }E\left[ {\parallel G_{d3} \left( {G_{e} \left( {I_{s} ,I_{f3} } \right) - I_{F3} } \right)\parallel_{1} } \right]$$

Feature-matching loss ${L}_{\mathrm{FM}}$ determines the difference between the generated and real FA images passing through an intermediate feature layer of the discriminator as follows [43]:

11 $$L_{{{\text{FM}}}} = E[\parallel D_{i,j} \left( {G_{d1} \left( {G_{e} \left( {I_{s} ,I_{f1} } \right)} \right)} \right) - D_{i,j} \left( {I_{F1} } \right)\parallel_{2}^{2} ]\, + \,E[\parallel D_{i,j} \left( {G_{d2} \left( {G_{e} \left( {I_{s} ,I_{f2} } \right)} \right)} \right) - D_{i,j} \left( {I_{F2} } \right)\parallel_{2}^{2} ]\, + \,E[\parallel D_{i,j} \left( {G_{d3} \left( {G_{e} \left( {I_{s} ,I_{f3} } \right)} \right)} \right) - D_{i,j} \left( {I_{F3} } \right)\parallel_{2}^{2} ]$$

Perceptual loss ${L}_{\mathrm{percep}}$ determines the difference between the generated and real FA images passing through an intermediate feature layer of the VGG19 network [44], thus allowing the generated FA image to retain deep semantic information [45]. It can be expressed as

12 $$L_{{{\text{percep}}}} = E[\parallel \varphi_{i,j} \left( {G_{d1} \left( {G_{e} \left( {I_{s} ,I_{f1} } \right)} \right)} \right) - \varphi_{i,j} \left( {I_{F1} } \right)\parallel_{2}^{2} ]\, + \,E[\parallel \varphi_{i,j} \left( {G_{d2} \left( {G_{e} \left( {I_{s} ,I_{f2} } \right)} \right)} \right) - \varphi_{i,j} \left( {I_{F2} } \right)\parallel_{2}^{2} ]\, + \,E[\parallel \varphi_{i,j} \left( {G_{d3} \left( {G_{e} \left( {I_{s} ,I_{f3} } \right)} \right)} \right) - \varphi_{i,j} \left( {I_{F3} } \right)\parallel_{2}^{2} ]$$

## Supplementary Information

**Additional file 1.** The table on the training procedure of HrGAN.

## Data Availability

The data sets used or analyzed during the current study are available from the corresponding author on reasonable request.

## Abbreviations

FA: Fundus fluorescein angiography
GAN: Generative adversarial network
LrGAN: Low-resolution GAN
HrGAN: High-resolution GAN
PSNR: Peak signal-to-noise ratio
SSIM: Structural similarity index
NCC: Cross-correlation correlation
Res: Residual attention block
FM: Feature matching
Percep: Perceptual
