# Supplementary material for: Synthesizing multi-frame high-resolution fluorescein angiography images from retinal fundus images using generative adversarial networks
Source: Biomed Eng Online. 2023 Feb 21;22:16. doi: 10.1186/s12938-023-01070-6 (PMC9945680; doi:10.1186/s12938-023-01070-6)
Supplement: Supplementary file 1 — Additional file 1. The table on the training procedure of HrGAN. [file 12938_2023_1070_MOESM1_ESM.docx]

| **Algorithm 1** Training procedure of HrGAN |
| --- |
| 1. Initialize the parameters of the shard encoder $G_{e}$: $\theta_{e}$  2. Initialize the parameters of the decoder $G_{d1}$: $\theta_{d1}$  3. Initialize the parameters of the decoder $G_{d2}$: $\theta_{d2}$  4. Initialize the parameters of the decoder $G_{d3}$*:* $\theta_{d3}$  5. Initialize the parameters of the discriminator $D_{1}$*:* $\theta_{D1}$  6. Initialize the parameters of the discriminator $D_{2}$*:* $\theta_{D2}$  7. Initialize the parameters of the discriminator $D_{3}$*:* $\theta_{D3}$  8. **repeat**  9. Sample a batch of fundus images $I_{s}\sim p(I_{s})$ and the corresponding low-resolution FA images $I_{f}\sim p(I_{f})$ generated by LrGAN.  10. Sample a batch of labeled transit-phase FA images $F1\sim p(F1).$  11. Generate pseudo transit-phase FA images $G_{d1}(G_{e}(I_{s},I_{f}))$.  12. Update $D_{1},G_{e}$ and $G_{d1}$ by ascending along its stochastic gradient:  $L_{D1}=\alpha E\left[ \log D_{1}\left( I_{F1} \right) \right]+\alpha E[log(1-D_{1}(G_{d1}(G_{e}(I_{s},I_{f}))))]$  $\theta_{D1}=\theta_{D1}-{\nabla_{\theta_{D1}}L}_{D1}$  $L_{e,d1}=E[\parallel G_{d1}(G_{e}(I_{s},I_{f})-I_{F1})\parallel_{1}]$*+*$E[\parallel D_{i,j}(G_{d1}(G_{e}(I_{s},I_{f})))-D_{i,j}(I_{F1})\parallel_{2}^{2}$]  +$E[\parallel\varphi_{i,j}(G_{d1}(G_{e}(I_{s},I_{f})))-\varphi_{i,j}(I_{F1})\parallel_{2}^{2}$]  $\theta_{d1}=\theta_{d1}{-\nabla}_{\theta_{d1}}L_{e,d1}$  $\theta_{e}=\theta_{e}-\nabla_{\theta_{e}}L_{e,d1}$  12. Sample a batch of labeled recirculation-phase FA images $F2\sim p(F2).$  13. Generate pseudo recirculation-phase FA images $G_{d2}(G_{e}(I_{s},I_{f}))$.  15. Update $D_{2},G_{e}$ and $G_{d2}$ by ascending along its stochastic gradient:  $L_{D2}=\alpha E\left[ \log D_{2}\left( I_{F2} \right) \right]+\alpha E[log(1-D_{2}(G_{d2}(G_{e}(I_{s},I_{f}))))]$  $\theta_{D2}=\theta_{D2}-{\nabla_{\theta_{D2}}L}_{D2}$  $L_{e,d2}=E[\parallel G_{d2}(G_{e}(I_{s},I_{f})-I_{F2})\parallel_{1}]$*+*$E[\parallel D_{i,j}(G_{d2}(G_{e}(I_{s},I_{f})))-D_{i,j}(I_{F2})\parallel_{2}^{2}$]  +$E[\parallel\varphi_{i,j}(G_{d2}(G_{e}(I_{s},I_{f})))-\varphi_{i,j}(I_{F2})\parallel_{2}^{2}$]  $\theta_{d2}=\theta_{d2}{-\nabla}_{\theta_{d2}}L_{e,d2}$  $\theta_{e}=\theta_{e}-\nabla_{\theta_{e}}L_{e,d2}$  16. Sample a batch of labeled late-phase FA images $F3\sim p(F3).$  17. Generate pseudo late-phase FA images $G_{d3}(G_{e}(I_{s},I_{f}))$.  18. Update $D_{3},G_{e}$ and $G_{d3}$ by ascending along its stochastic gradient:  $L_{D3}=\alpha E\left[ \log D_{3}\left( I_{F1} \right) \right]+\alpha E[log(1-D_{3}(G_{d3}(G_{e}(I_{s},I_{f}))))]$  $\theta_{D3}=\theta_{D3}-{\nabla_{\theta_{D3}}L}_{D3}$  $L_{e,d3}=E[\parallel G_{d3}(G_{e}(I_{s},I_{f})-I_{F3})\parallel_{1}]$*+*$E[\parallel D_{i,j}(G_{d3}(G_{e}(I_{s},I_{f})))-D_{i,j}(I_{F3})\parallel_{2}^{2}$]  +$E[\parallel\varphi_{i,j}(G_{d3}(G_{e}(I_{s},I_{f})))-\varphi_{i,j}(I_{F3})\parallel_{2}^{2}$]  $\theta_{d3}=\theta_{d1}{-\nabla}_{\theta_{d3}}L_{e,d3}$  $\theta_{e}=\theta_{e}-\nabla_{\theta_{e}}L_{e,d3}$  19. **until** convergence  20. **return** $\theta_{e,} \theta_{d1,}\theta_{d2,}\theta_{d3,}\theta_{D1,}\theta_{D2,} \theta_{D3}$ |
